# Examining Personalities and Behavioural Syndromes in the Burying Beetle, *Nicrophorus vespilloides* Herbst, 1783

**DOI:** 10.1002/ece3.71718

**Published:** 2025-07-04

**Authors:** Pavol Prokop, Jozef Balcečík, Rudolf Masarovič, Zuzana Provazník

**Affiliations:** ^1^ Institute of Zoology Slovak Academy of Sciences Bratislava Slovakia; ^2^ Department of Environmental Ecology and Landscape Management Faculty of Natural Sciences, Comenius University Bratislava Slovakia

**Keywords:** insect mobility, physical condition, stridulation, tonic immobility

## Abstract

Animal personality encompasses behavioral traits that can vary between individuals while remaining stable over time for each individual. These traits often correlate with each other and form behavioral syndromes. In our study, we investigated the personalities and behavioral syndromes of the burying beetle *Nicrophorus vespilloides*, which defends and prepares carcasses as food for its offspring. In the laboratory, we repeatedly examined overall mobility, explorative behavior, duration of stridulation, and tonic immobility in the same individuals. Overall mobility and stridulation exhibited moderate repeatability in both sexes. Tonic immobility (TI) was more repeatable in females than in males, whereas exploratory behavior did not show repeatability in either sex. Results showed that males remained in tonic immobility for significantly longer periods than females. Contrary to our expectations, females exhibited greater exploratory behavior than males. The duration of stridulation was similar between the sexes, but individuals with poorer body conditions stridulated for a longer time than individuals with better physical conditions. Stridulation is triggered by simulated predatory attacks, suggesting that it may serve as a defense mechanism against predators. We conclude that the functional significance of personality traits in *N. vespilloides* warrants further investigation in the context of their natural predators and conspecifics.

## Graphical abstract

*Nicrophorus vespilloides* personalities are almost unknown. Moreover, the functional significance of stridulation in burying beetles is poorly understood. We examined personality traits and behavioural syndromes in these species concerning sex differences and body condition.

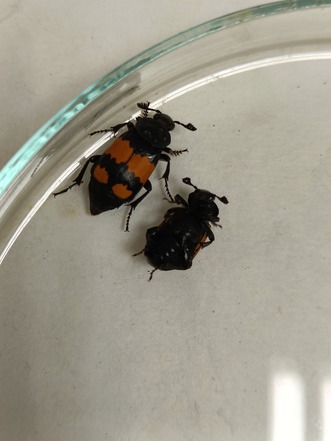

## 1 Introduction

Anti‐predator behaviors are critical for survival and reproductive success across animal taxa (Huang and Caro 2023). These behaviors often involve trade‐offs between risk‐taking and safety, with individuals balancing the need to acquire resources against the potential costs of predation (Clark 1994; Sih and Del Giudice 2012). For example, some species and individuals employ active defences, such as aggression or sound production, while others rely on passive strategies, such as immobility or camouflage, to evade detection (Ruxton et al. 2019; Carli and Farabollini 2022).

Such individual variation in the expression of anti‐predator behaviors is not always random; rather, it can be attributed to consistent differences among individuals, often described as animal personality. Personality traits represent consistent styles of behavioral responses to various stimuli (Gosling 2001; Bell and Aubin‐Horth 2010). These traits are assessed in terms of their repeatability and stability over time, indicating that an individual's personality remains consistent across different contexts (Bell and Aubin‐Horth 2010; Cabrera et al. 2021). Correlations between personality traits suggest that certain traits may be part of a broader behavioral syndrome, defined as a suite of correlated behaviors that manifest across different contexts or situations within a population (Briffa and Weiss 2010; Sih et al. 2004; Sih and Del Giudice 2012). Research has documented animal personality in a wide range of species, including invertebrates (Cabrera et al. 2021).

Certain behavioral traits, such as boldness and aggression, can significantly influence an individual's anti‐predator strategies. For instance, bolder individuals may engage in riskier behaviors, such as foraging in exposed areas, which can enhance their access to resources and reproductive success but also increase their vulnerability to predators (Smith and Blumstein 2008; Eccard et al. 2022). More cautious individuals may display behaviors that prioritize safety, including seeking refuge or using tactics like mimicry or tonic immobility when threatened (Dingemanse et al. 2004; Sih et al. 2004). Longer durations of tonic immobility were found to be associated with food deprivation (Taylor et al. 2023) and with lower activity levels (Matsumura and Miyatake 2019). Bolder individuals also tend to employ more aggressive and proactive strategies when faced with threats; they habituate more quickly to novel environments and exhibit lower levels of anxiety, along with a greater propensity for exploration (Goodchild et al. 2020; Eccard et al. 2022; Bibi et al. 2023) and mobility (Schirmer et al. 2019).

Beetles of the genus *Nicrophorus* (Coleoptera: Silphidae) utilize carrions of small vertebrates, such as songbirds or mice, for breeding (Scott 1998; Potticary et al. 2024). These beetles prepare carcasses by defending, burying, removing hair, and impregnating the remains with antimicrobial substances to prevent decay and protect their larvae (Rozen et al. 2008; Hall et al. 2011). Burying the carcass can be considered an anti‐predator tactic that conceals it from larger vertebrate scavengers and aerial predators. After hatching, the larvae migrate to the carcass, where they receive biparental care. Both parents provision their offspring with predigested carrion through oral trophallaxis and protect the brood from predators, conspecifics, microbes, and competing insects (Scott 1998; Meierhofer et al. 1999; Malik et al. 2024). Approximately 1 week after hatching, the larvae complete their development and disperse from the carcass remnants to pupate in the soil, while the adult beetles depart in search of new breeding opportunities (Scott 1998).

Parental care in *Nicrophorus* spp. also involves distinct anti‐predator behaviors, such as tonic immobility and stridulation, which can serve to protect themselves and/or directly safeguard the offspring or the prepared carcass. Tonic immobility, a state of temporary paralysis exhibited by parent beetles, is believed to reduce the likelihood of predator detection when active resistance or escape is no longer feasible (Humphreys and Ruxton 2018). This behavior is common in *N. vespilloides* , particularly when individuals are physically handled, grasped, or face an immediate threat. Stridulation, on the other hand, involves sound production by rubbing the abdomen against the elytra (Hall et al. 2015; Phillips et al. 2020; Conrad et al. 2024). Although stridulation was once thought to play a role in parental care, recent evidence suggests it primarily serves an antipredator function, similar to that observed in other beetles (Hall et al. 2013; Schrader and Galanek 2022; Lewis and Cane 1990). Both tonic immobility and stridulation are primarily forms of self‐defense for the parents, but both are also exhibited during the protection of larvae or resource foraging. In natural settings, males tend to desert broods earlier than females (Eggert and Müller 1997; Ward et al. 2009), and females spend more time provisioning food than males (Smiseth and Moore 2004; Walling et al. 2008), indicating that males invest less in parental care than do females. Consequently, males may be more inclined to explore their environment to search for new mating opportunities. However, this behavior also exposes them to greater risks, particularly when defending carcasses against both conspecific and heterospecific competitors.

Our objective was to determine whether tonic immobility, stridulation, exploratory behavior, and mobility are repeatable personality traits in the burying beetle *Nicrophorus vespilloides* . Exploratory behavior is a frequently used component of personality (Dingemanse et al. 2002; Van Oers et al. 2005). Research across species, including sticklebacks, convict cichlids, and Great Tits, indicates that individuals exhibiting higher levels of exploration tend to be bolder (Bell 2005; Jones and Godin 2010; Bibi et al. 2023). We also examined whether these personality traits were correlated. Examining correlations among these personality traits allows us to assess the presence of behavioral syndromes—groups of correlated behaviors that consistently appear together across different contexts or situations within a population (Briffa and Weiss 2010; Sih et al. 2004; Sih and Del Giudice 2012). We hypothesize that individuals exhibiting higher exploratory behavior, and therefore bolder personalities, are more likely to engage in stridulation as an active antipredator response and to display reduced tonic immobility (Carli and Farabollini 2022). Furthermore, we expect that more explorative individuals will demonstrate greater mobility than less explorative individuals (Koski 2014; Schirmer et al. 2019). Given that animals experiencing hunger often engage in riskier behaviors to find food, we expected beetles in poorer physical condition to exhibit higher mobility (Moran et al. 2021). Furthermore, we expected good physical condition in *N. vespilloides* to be negatively correlated with the duration of tonic immobility (Taylor et al. 2023) and positively correlated with stridulation, given that body condition is positively associated with active antipredator behavior in animals (Bachmann 1993; Kenward 1978). Finally, we examined the potential differences in personality traits between males and females. We hypothesize that males are likely to exhibit higher levels of exploration, greater mobility, longer durations of stridulation, and longer tonic immobility than females.

## 2 Methods

### 2.1 Rearing Conditions

The stock population of *N. vespilloides* used for the experiments was established with 50 pairs of beetles captured from two sites in Western Slovakia (48°17′10.86″ N, 17°38′56.72″ E). They were interbred in the laboratory for two generations before the start of the experiment. Adult beetles were kept in single‐sex plastic boxes (25 × 16 × 13 cm) filled with moist peat at a constant temperature of 19°C and a light cycle of 16:8 and fed twice weekly with thawed chicken hearts, thawed chicken liver, or freshly killed mealworms before mating (e.g., Smiseth et al. 2008). Mealworms were humanely euthanized by freezing at −18°C to halt metabolic processes, and then subsequently cut into pieces.

### 2.2 Breeding

The breeding was established in 0.7‐L glass jars, approximately 12 cm in height and with an opening diameter of 6 cm, which were covered at the top with a dense fabric that ensured adequate ventilation while preventing the beetles from escaping into the laboratory environment. Relative humidity in the laboratory was maintained at 75% (±10%) (Comet Logger s3120). The jars were filled with moist peat up to two‐thirds full and then placed on a thawed carcass of a mouse (Wang et al. 2021). Commercially obtained mice were approximately 10 cm long and weighed between 25 and 30 g. The jars were checked approximately 24 h later. If the mouse was successfully buried, we did not further manipulate the jar but maintained the appropriate moisture level of the peat. We only monitored the surface moisture of the peat daily and maintained the appropriate humidity by lightly spraying water whenever the surface appeared dry. Five days after the establishment of breeding on *N. vespilloides* , we emptied the contents of the jar and removed the adult individuals. If they are left too long without food, they could attack their larvae. We then returned the contents of the jar, together with the larvae and carcass, so that the larvae could complete their development to the imago stage.

### 2.3 Procedure

For the experiment, we used virgin adult individuals, approximately 14 days old, *N* = 39 males and *N* = 38 females, taken from the breeding set‐up described above. Each individual was housed separately in a container of height, filled with peat up to two‐thirds. Feeding occurred 2–3 times a week, with approximately one‐third of the chicken hearts thawed. Before the start of the experiment, each beetle was weighed on an electronic balance ABS 120‐4 N (to the nearest 0.1 mg), and its pronotum width was measured using a digital calliper (KINEX/K‐MET Absolute Zero 150/40/0.01 mm). All behavioral assays were conducted on both sexes, and data were analyzed separately for males and females to account for potential sex differences.

### 2.4 Measuring Tonic Immobility and Stridulation

We placed the individual test beetle of both sexes on a hard plastic surface (30 × 30 cm), ventral side up, and gently tapped its ventral part with soft entomological forceps to simulate predation, such as a peck from a bird that it might experience in the wild (Lindstedt et al. 2017). Similar tactile stimuli triggering tonic immobility are widely accepted, non‐lethal methods to induce antipredator responses in insects, allowing standardized and repeatable assessment of behavioral reactions under controlled laboratory conditions (e.g., using a woody stick as in Matsumura 2025). Each beetle was tested three times. We recorded the following behaviors using a stopwatch:

Stridulation duration (s).

Duration of tonic immobility (TI) (s).

Stridulation (if any) always preceded TI; thus, these two behaviors did not interfere. Behavioral recording was continued until the behavior ceased. After the experiment, each individual was placed in a housing cage.

### 2.5 Measuring Mobility and Exploration

Mobility and exploration were assessed using video recordings of beetles in a circular arena. We used freely available software, Buridan Tracker (https://buridan.sourceforge.net) (Götz 1980). This software is designed to record the mobility of the individual in the arena. The arena was circular, with a diameter of 15 cm and a height of 2 cm (Figure S1). It was illuminated with red light, which simulates darkness for the beetles, an important factor since their activity is nocturnal. The entire experiment was recorded with a camera positioned 35 cm above the arena. Each tested individual was briefly anaesthetized with CO_2_ (for a maximum of 1 min) and placed ventral side down in the center of the arena. As soon as the beetle showed the first signs of activity, we started recording, and the test was considered initiated. The observation period lasted 5 min. During this time, we measured beetle mobility, quantified as the total distance traveled (mm). Exploratory behavior was defined as the proportion of time spent within a 10 cm diameter circle centered in the arena. Bold individuals are more likely to traverse the center of the arena, while shy individuals tend to stay near the edges, where they are protected by the solid border. These measures were chosen to capture distinct aspects of beetle behavior, with mobility reflecting overall activity levels and exploration reflecting boldness within the arena. Our classification is based on Réale et al. (2007), who suggest that bold individuals tend to explore open spaces (e.g., the center of an arena), whereas shy individuals display wall‐hugging behavior due to heightened anxiety or risk aversion. Measurements were repeated for each individual, with a maximum of one test per day to avoid habituation or exhaustion, ensuring that all tests were completed within 6 days.

### 2.6 Measuring Repeatability

The repeatability (*r*) of certain behaviors is considered a crucial first step in exploring the genetic basis of behavior (Dohm 2002; van Oers et al. 2004). This implies that low within‐individual repeatability may result from various non‐genetic factors. To calculate the repeatability (*r*) of stridulation duration, tonic immobility duration, mobility, and exploration, we followed the methods of Lessells and Boag (1987) using intraclass correlation coefficients, which represent the proportion of variance in a trait attributable to differences among individuals relative to the total variance. The repeatability values range from 0 to 1, where 0 indicates no repeatability and 1 indicates perfect repeatability. To interpret the strength of repeatability, we followed the thresholds proposed by Wolak et al. (2012): low repeatability (*r* < 0.20), intermediate repeatability (0.30 ≤ *r* < 0.6), and high repeatability (*r* ≥ 0.7).

### 2.7 Measuring Physical Condition

Individual body condition was calculated as residuals of regression between body mass, controlled for pronotum width (Jakob et al. 1996).

### 2.8 Statistical Analyses

Data on stridulation, TI, exploration, and mobility were not normally distributed, and their distributions could not be improved through various methods (e.g., Box‐Cox transformation, logarithmic transformation). We therefore used the Poisson distribution of dependent variables in generalized linear mixed models (GLMM). The full model for each personality trait was:

Trait ~ Sex + Round + Body condition + Sex × Round + Sex × Body condition + Round × Body condition + Sex × Round × Body condition + (1|ID). We fitted a full generalized linear mixed model (GLMM) including all predictors (sex, round, body condition) and their interactions, as all interactions were significant. The significance of predictors and interactions was assessed using likelihood ratio tests (LRTs). Simple correlations among the mean values calculated for each personality trait were performed with Spearman rank correlation coefficients. All tests were performed with the Jamovi software (The Jamovi Project 2023).

## 3 Results

### 3.1 Correlations Between Pronotum and Body Mass

To understand the relationship between morphological traits and body size, we examined correlations between pronotum width and body mass in the tested beetles. Pronotum width was strongly correlated with body mass in both males (*r* = 0.89, *p* < 0.001, *N* = 39) and females (*r* = 0.93, *p* < 0.001, *N* = 38).

### 3.2 Personality Traits

We measured personality traits, including stridulation, tonic immobility, mobility, and exploration in a sample of *N* = 39 males and *N* = 38 females. All behavioral assays were conducted separately for each sex to examine potential sex differences. Descriptive data are shown in Table 1.

**TABLE 1:** Mean data for behavioral and morphometric tests of males (*N* = 39) and females (*N* = 38).

	Sex	Mass (g)	Pronotum width (mm)	Stridulation (s)	Tonic immobility (s)	Exploration (%)	Mobility (mm)
Mean	Male	0.261	5.29	5.29	192	12.5	1900
	Female	0.26	5.26	6.08	101	28.2	2447
SE	Male	0.0066	0.0536	1.33	21	2.71	115
	Female	0.00785	0.0649	1.98	13.9	3.96	212
Minimum	Male	0.155	4.35	0	4	0.583	672
	Female	0.119	4.12	0	0	3.72	817
Maximum	Male	0.36	6.07	32.7	599	97.1	3657
	Female	0.333	5.79	54.7	391	125	6115

Stridulation, TI and overall mobility received statistically significant repeatability, but much higher in females, and exploration received low repeatability in both sexes (Table 2).

**TABLE 2:** Repeatability of behavior was calculated for males and females of *N. vespilloides*.

	Males			Females		
	*F* (38,78)	*r*	*p*	*F* (37,76)	*r*	*p*
Stridulation	2.97	0.39	< 0.001	2.94	0.39	< 0.001
Tonic immobility	1.78	0.21	0.02	2.37	0.31	< 0.001
Overall mobility	2.16	0.29	0.002	3.81	0.48	< 0.001
Exploration	1.16	0.05	0.28	1.44	0.13	0.09

### 3.3 Behavioral Syndromes

Behavioral traits did not show significant correlations with each other (Table 3). An exception was TI, which moderately and negatively correlated with exploration (Figure S2). This suggests that more explorative individuals tended to remain in TI for a shorter time than less explorative individuals. Note that controlling for the effect of sex did not change the results of the statistical analyses.

**TABLE 3:** Spearman rank correlation coefficients for behavioral data.

Measure	Spearman's rho	Stridulation	Tonic immobility	Explorative behavior
Tonic immobility	rho	−0.116	—	
	*p*	0.314	—	
Explorative behavior	rho	−0.019	−0.247	—
	*p*	0.866	0.030	—
Mobility	rho	0.067	0.031	0.176
	*p*	0.565	0.790	0.126

Furthermore, we analysed possible differences between males and females and whether individual biometry influences personality traits. The mean values are shown in Table 1.

#### 3.3.1 Stridulation

**FIGURE 1:**
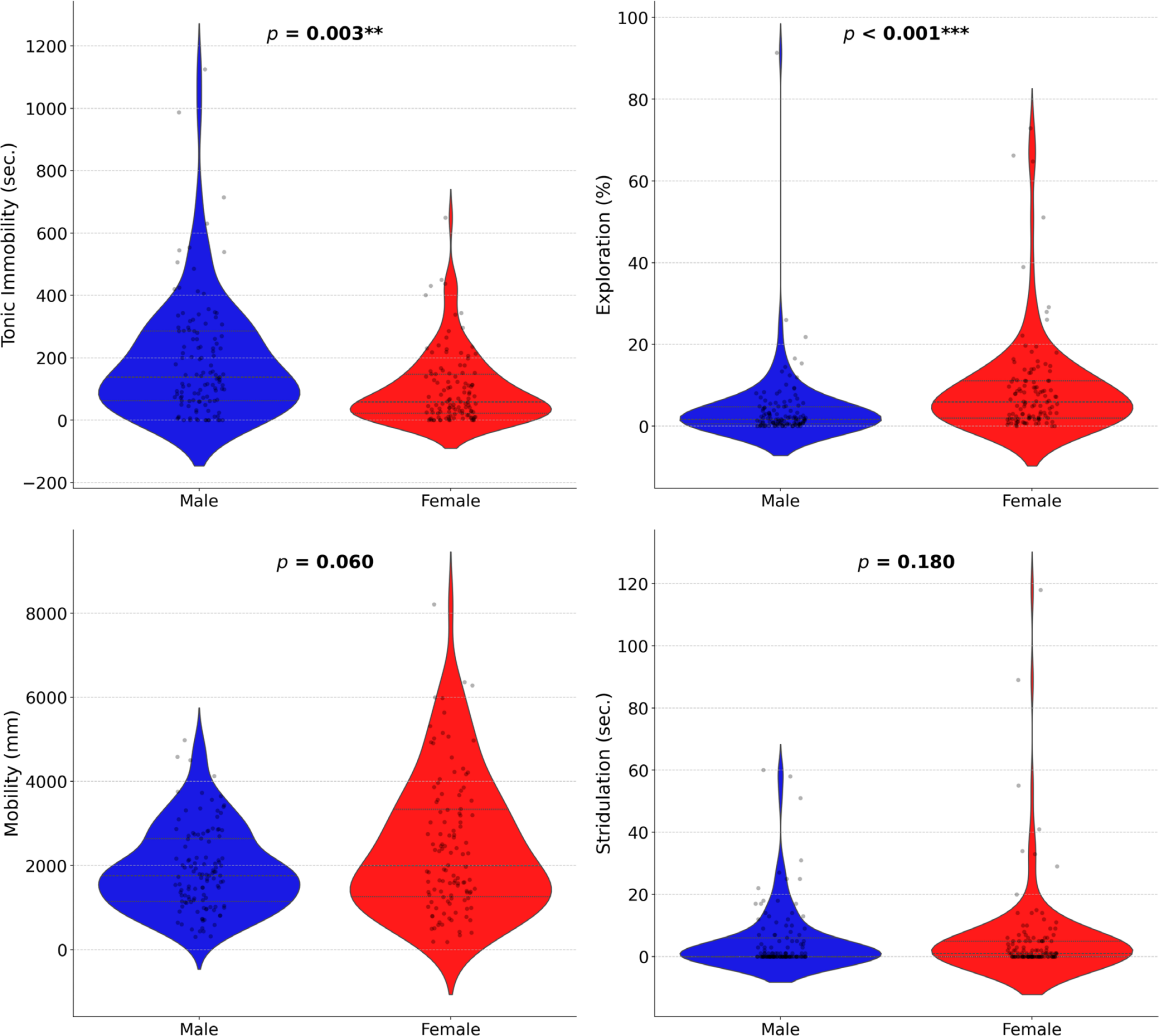
Sex differences in behavioral traits of *N. vespilloides*.

Stridulation was not significantly influenced by sex (Table 4, Figure 1). There was a negative influence of body condition on stridulation (Table S1). Females stridulated longer in the first round, and the stridulation duration decreased during subsequent testing. In contrast, males tended to stridulate most in the second round and less in the first and third rounds (Figure S3).

**TABLE 4:** Results of GLMMs exploring the impact of sex and body condition on personality traits in *Nicrophorus vespilloides* .

Effect on	Variable	df	Test statistic *χ* ^2^	*p*
Stridulation	Whole model	7	2426.138	< 0.001
	Sex (F‐M)	1	1.82	0.177
	Body condition	1	4.84	0.028
	Round	2	38.39	< 0.001
	Sex × Round	2	24.45	< 0.001
Tonic immobility	Whole model	7	20,810.23	< 0.001
	Sex	1	9.08	0.003
	Body condition	1	3.16	0.076
	Round	2	30.28	< 0.001
	Sex × Round	2	442.43	< 0.001
Mobility	Whole model	7	115,009.927	< 0.001
	Sex	1	3.45	0.063
	Body condition	1	1.74	0.188
	Round	2	1185.29	< 0.001
	Sex × Round	2	2215.2	< 0.001
Exploration	Whole model	7	1242.892	< 0.001
	Sex	1	24.793	< 0.001
	Body condition	1	0.493	0.482
	Round	2	8.805	0.012
	Sex × Round	2	13.592	0.001

#### 3.3.2 Tonic Immobility

Males were more prone to TI than females, but body condition did not influence TI (Table 4, Table S2). Males in the third round prolonged TI, while females decreased the time spent in TI (Figure S4).

#### 3.3.3 Mobility

Mobility was not significantly influenced by beetles' sex or body condition (Table 4). Females were more mobile than males in the first round, but mobility in the second and third rounds was similar between the sexes (Figure S5). Further statistical details are in Table S3.

#### 3.3.4 Exploration

Females were significantly more explorative than males. Body condition did not influence the explorative behavior of beetles (Table 4, Table S4). While females showed consistent explorative behavior across repeated measures, males were less explorative in the first round (Figure S6).

## 4 Discussion

We found that stridulation and mobility are repeatable behavioral traits that can be considered as personality indicators in *N. vespilloides* . Tonic immobility (TI) appears to be sex‐specific; this trait was more repeatable in females than in males, and it took significantly longer in males. We observed a negative correlation solely between TI and exploratory behavior. Further discussions of sex‐specific associations and body conditions are presented below.

### 4.1 Associations Among Behavioral Variables

We expected a negative correlation between physical condition and beetle mobility (Moran et al. 2021). Mobility in *N. vespilloides* was repeatable, as it was found in other arthropods (Prokop and Semelbauer 2017; Tanaka 2009). This behavioral trait is crucial for *N. vespilloides* when searching for food sources because there is strong competition for carcasses in nature (Scott et al. 1987; Trumbo 1990). Successful monopolization of a carcass ultimately determines the reproductive success of burying beetles (Scott 1998). The beetle movement was not related to their physical condition, which does not support our hypothesis. An alternative explanation is that the lack of a negative correlation between mobility and physical condition may explain the dominant role of physical traits in competitive success rather than behavioral traits like mobility. Our results did not support the idea that beetles in better physical condition would spend less time in tonic immobility. In fact, we found that beetles in better condition spent less time stridulating, which was unexpected. Overall, this means there is no clear positive link between body condition and defensive behavior in *N. vespilloides* (Bachmann 1993; Kenward 1978). One possible explanation for this finding is that TI is a more passive anti‐predator strategy than the fight or flight response (Matsumura and Miyatake 2019; Carli and Farabollini 2022); thus, it does not need to be associated with an individual's physical condition. TI was negatively correlated with exploration, again supporting the idea that TI may be a passive defensive strategy that is more common among individuals with shyer personalities. Matsumura and Miyatake (2019) similarly found that longer durations of tonic immobility were associated with lower activity levels in the red flour beetle, *Tribolium castaneum* . Correlations among personality traits suggest that these traits may be part of a broader behavioral syndrome, defined as a suite of correlated behaviors that manifest across different contexts or situations within a population (Briffa and Weiss 2010; Sih et al. 2004; Sih and Del Giudice 2012). It is important to note that the mobility tests were conducted in the absence of predatory signals, unlike TI tests. Predatory signals are known to influence arthropod mobility (Binz et al. 2014; de Heij et al. 2023). Therefore, the context in which TI was assessed may have influenced the outcomes, making it difficult to directly compare the results of the mobility tests conducted without predatory cues.

Audible stridulation in burying beetles is common in various contexts, including courtship, carcass preparation, and parental care (Pukowski 1933; Phillips et al. 2020), yet its significance remains uncertain (Schrader and Galanek 2022). The repeatability of this trait clearly supports the idea that stridulation is an important part of the behavioral repertoire of *N. vespilloides* , and its activation during simulated predation suggests that it serves an antipredatory function (Lewis and Cane 1990).

### 4.2 Differences Between Sexes

We found no evidence that males were more exploratory, mobile, or spent more time stridulating than females. Although males showed longer durations of tonic immobility, this contradicts our initial hypotheses. Typically, males of many species display riskier and bolder behaviors during mating to attract females and increase their chances of mating (Dingemanse and Réale 2005). Defending carcasses against competitors means that males may be more frequently exposed to predators than females are. Males can adopt tonic immobility when faced with perceived threats to avoid injury or death, which can reduce their reproductive success. Therefore, a longer TI as an anti‐predator strategy might be more advantageous for males than for females. Interestingly, TI showed high intraspecific variability; some individuals did not exhibit TI, whereas others remained in this state for more than 18 min. The duration of stridulation did not differ between males and females, indicating that this form of communication is similarly used by both sexes of *N. vespilloides* when interacting with conspecifics (Pukowski 1933; Phillips et al. 2020; Schrader and Galanek 2022). However, it is unclear why females exhibit greater exploration than males. One possible explanation is that females, who typically provide more care for their offspring than males (Smiseth and Moore 2004; Walling et al. 2008) may compensate for the energy expended in nurturing by engaging in more extensive foraging activities. Alternatively, females may display higher exploratory behavior due to their role in locating and preparing food for their offspring (Potticary et al. 2024). Research has shown that parental care in *N. vespilloides* is costly for females (Smiseth and Moore 2004; Walling et al. 2008; Cotter et al. 2010; Ward et al. 2009); therefore, this possibility cannot be ruled out. Lindstedt et al. (2017), for instance, found that female *N. vespilloides* produce a greater volume of antimicrobial anal fluid with higher lytic activity than males. As they suggest, this could be related to differences in parental investment and defense strategies. Since females spend more time attending to the carcass and may face a greater risk of predation, they might secrete more exudate as a defensive response.

## 5 Conclusion

In conclusion, stridulation and overall mobility appear to be reliable behavioral traits of *N. vespilloides* . Although tonic immobility (TI) is less repeatable in males, it remains an important trait that exhibits significant variability and sex specificity. More research is needed to explore the functional significance of stridulation and TI in encounters between beetles and their natural predators.

## Author Contributions

**Pavol Prokop:** conceptualization (equal), formal analysis (equal), funding acquisition (equal), investigation (equal), methodology (equal), supervision (equal), writing – original draft (equal), writing – review and editing (equal). **Jozef Balcečík:** conceptualization (equal), data curation (equal), investigation (equal), methodology (equal), software (equal), visualization (equal), writing – review and editing (equal). **Rudolf Masarovič:** conceptualization (equal), investigation (equal), methodology (equal), writing – review and editing (equal). **Zuzana Provazník:** data curation (equal), investigation (equal), methodology (equal), writing – review and editing (equal).

## Conflicts of Interest

The authors declare no conflicts of interest.

## Supporting information


Appendix S1

Appendix S2

## Data Availability

The data are available as Appendices S1 and S2.
