# Supplementary material for: Examining Personalities and Behavioural Syndromes in the Burying Beetle, Nicrophorus vespilloides Herbst, 1783
Source: Ecol Evol. 2025 Jul 4;15(7):e71718. doi: 10.1002/ece3.71718 (PMC12231233; doi:10.1002/ece3.71718)

Supplementary Table 1. Parameter estimates for stridulation (dependent variable)

|  | | | | | **Exp(B) 95% Confidence Intervals** | |  | |
| --- | --- | --- | --- | --- | --- | --- | --- | --- |
| **Names** | **Effect** | **Estimate** | **SE** | **Exp(B)** | **Lower** | **Upper** | **z** | **p** |
| (Intercept) | (Intercept) | 0.193 | 0.257 | 1.213 | 0.733 | 2.0075 | 0.751 | 0.453 |
| Sex1 | Female - Male | 0.664 | 0.4915 | 1.942 | 0.741 | 5.0905 | 1.351 | 0.177 |
| Body condition | Body condition | -31.95 | 14.522 | 1.33E-14 | 5.79E-27 | 0.0306 | -2.2 | 0.028 |
| Round1 | 1.2 | -0.187 | 0.0654 | 0.83 | 0.73 | 0.9432 | -2.853 | 0.004 |
| Round2 | 2.3 | 0.432 | 0.0698 | 1.541 | 1.344 | 1.7664 | 6.19 | < .001 |
| Sex1 ✻ Round1 | (Female - Male) ✻ (1 - 2) | 0.641 | 0.1308 | 1.899 | 1.47 | 2.4543 | 4.903 | < .001 |
| Sex1 ✻ Round2 | (Female - Male) ✻ (2 - 3) | -0.369 | 0.1396 | 0.692 | 0.526 | 0.9092 | -2.642 | 0.008 |

Supplementary Table 2. Parameter estimates for tonic immobility (dependent variable)

|  | | | | | **Exp(B) 95% Confidence Intervals** | |  | |
| --- | --- | --- | --- | --- | --- | --- | --- | --- |
| **Names** | **Effect** | **Estimate** | **SE** | **Exp(B)** | **Lower** | **Upper** | **z** | **p** |
| (Intercept) | (Intercept) | 4.5629 | 0.1168 | 95.861 | 76.252 | 120.513 | 39.077 | < .001 |
| Sex1 | Female - Male | -0.70516 | 0.234 | 0.494 | 0.312 | 0.782 | -3.014 | 0.003 |
| Body condition | Body condition | -11.34214 | 6.3853 | 1.19E-05 | 4.36E-11 | 3.231 | -1.776 | 0.076 |
| Round1 | 1.2 | -0.06531 | 0.014 | 0.937 | 0.911 | 0.963 | -4.675 | < .001 |
| Round2 | 2.3 | -0.00422 | 0.014 | 0.996 | 0.969 | 1.024 | -0.3 | 0.764 |
| Sex1 ✻ Round1 | (Female - Male) ✻ (1 - 2) | -0.03414 | 0.0279 | 0.966 | 0.915 | 1.021 | -1.222 | 0.222 |
| Sex1 ✻ Round2 | (Female - Male) ✻ (2 - 3) | 0.53373 | 0.0281 | 1.705 | 1.614 | 1.802 | 19.006 | < .001 |

Supplementary Table 3. Parameter estimates for immobility (dependent variable)

|  | | | | | **Exp(B) 95% Confidence Intervals** | |  | |
| --- | --- | --- | --- | --- | --- | --- | --- | --- |
| **Names** | **Effect** | **Estimate** | **SE** | **Exp(B)** | **Lower** | **Upper** | **z** | **p** |
| (Intercept) | (Intercept) | 7.5677 | 0.05237 | 1934.6785 | 1745.932 | 2143.83 | 144.49 | < .001 |
| Sex1 | Female - Male | 0.195 | 0.10503 | 1.2153 | 0.989 | 1.493 | 1.86 | 0.063 |
| Body condition | Body condition | -3.8664 | 2.93507 | 0.0209 | 6.64E-05 | 6.594 | -1.32 | 0.188 |
| Round1 | 1.2 | 0.0637 | 0.00344 | 1.0658 | 1.059 | 1.073 | 18.51 | < .001 |
| Round2 | 2.3 | 0.0565 | 0.00354 | 1.0581 | 1.051 | 1.065 | 15.97 | < .001 |
| Sex1 ✻ Round1 | (Female - Male) ✻ (1 - 2) | 0.3222 | 0.00689 | 1.3801 | 1.362 | 1.399 | 46.78 | < .001 |
| Sex1 ✻ Round2 | (Female - Male) ✻ (2 - 3) | -0.1327 | 0.00707 | 0.8758 | 0.864 | 0.888 | -18.76 | < .001 |

Supplementary Table 4. Parameter estimates for exploration (dependent variable)

|  | | | | | **Exp(B) 95% Confidence Intervals** | |  | |
| --- | --- | --- | --- | --- | --- | --- | --- | --- |
| **Names** | **Effect** | **Estimate** | **SE** | **Exp(B)** | **Lower** | **Upper** | **z** | **p** |
| (Intercept) | (Intercept) | 1.2657 | 0.1197 | 3.546 | 2.804 | 4.483 | 10.571 | < .001 |
| Sex1 | Female - Male | 1.1856 | 0.2381 | 3.273 | 2.052 | 5.219 | 4.979 | < .001 |
| Body condition | Body condition | 4.6945 | 6.6828 | 109.339 | 2.24E-04 | 5.34E+07 | 0.702 | 0.482 |
| Round1 | 1.2 | -0.2088 | 0.0706 | 0.812 | 0.707 | 0.932 | -2.957 | 0.003 |
| Round2 | 2.3 | 0.0736 | 0.0673 | 1.076 | 0.943 | 1.228 | 1.094 | 0.274 |
| Sex1 ✻ Round1 | (Female - Male) ✻ (1 - 2) | 0.5023 | 0.1412 | 1.653 | 1.253 | 2.18 | 3.556 | < .001 |
| Sex1 ✻ Round2 | (Female - Male) ✻ (2 - 3) | -0.33 | 0.1346 | 0.719 | 0.552 | 0.936 | -2.452 | 0.014 |

Supplementary Figure 1. Construction of the arena: (a) a removable glass sheet covering the arena, which prevents the beetle from escaping and allows for placement of the beetle into the arena; (b) a plastic block delimiting the arena; (c) a removable glass sheet that is cleaned between each replication.


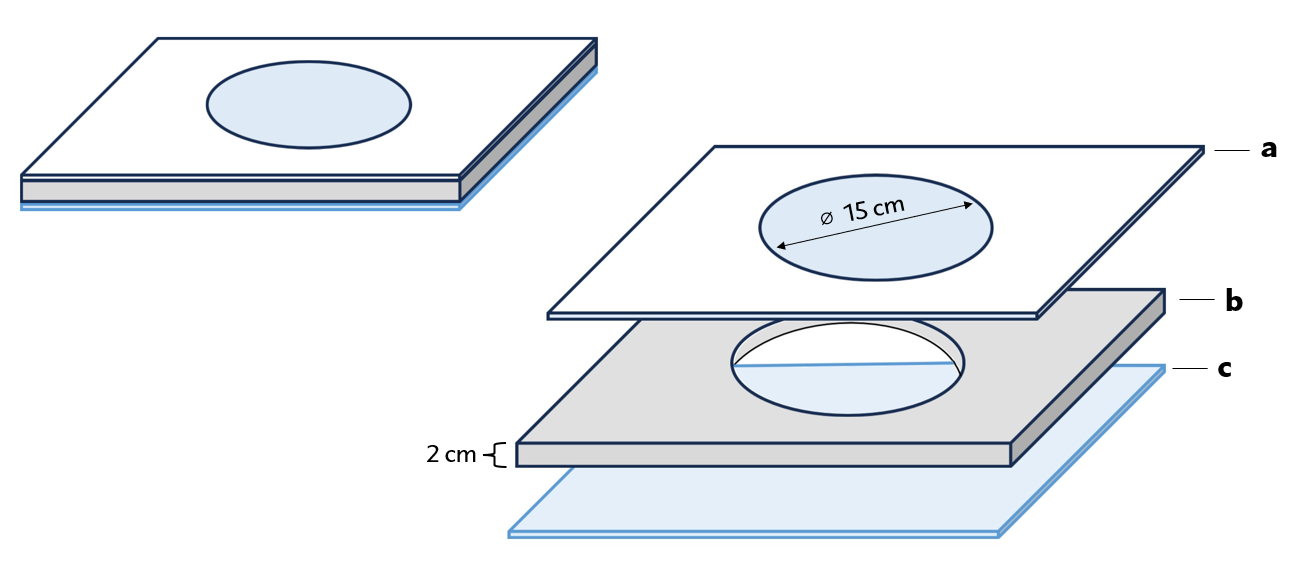


Supplementary Figure 2. Correlation between Exploration and TI in *N. vespilloides* males and females


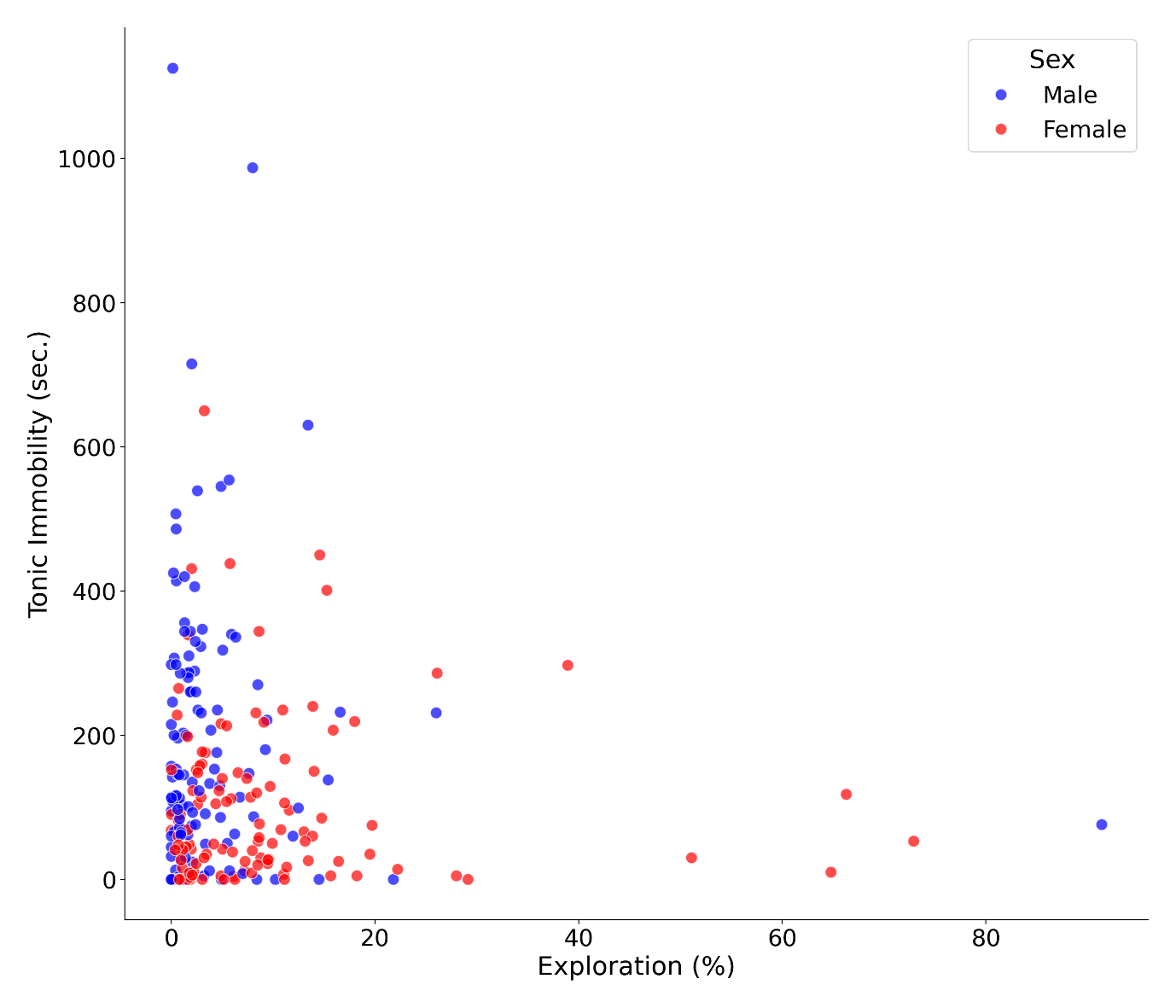


Supplementary Figure 3. Interaction effect between beetle sex and trial round on stridulation. Values represent means ± 95% confidence intervals (CI).


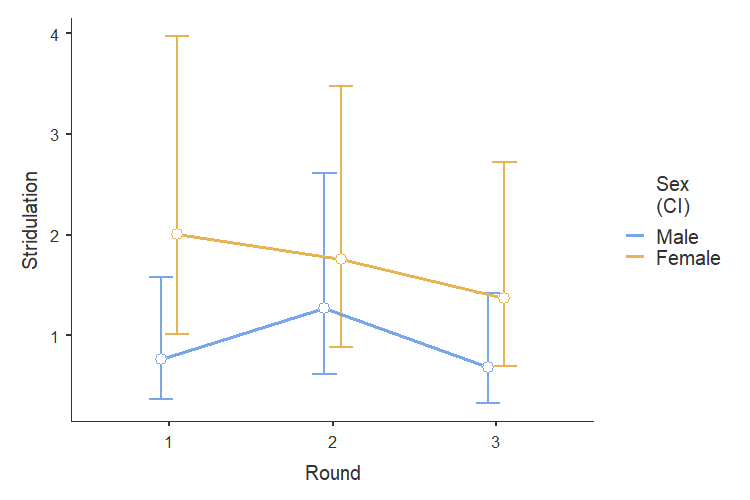


Supplementary Figure 4. Interaction effect between beetle sex and trial round on tonic immobility. Values represent means ± 95% confidence intervals (CI).


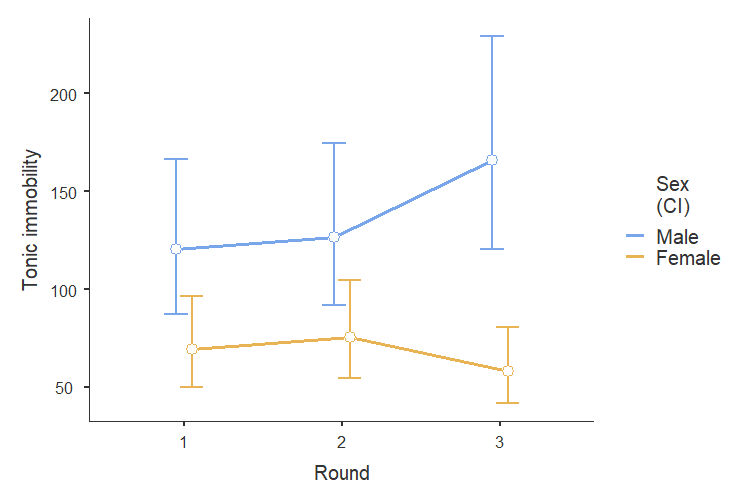


Supplementary Figure 5. Interaction effect between beetle sex and trial round on mobility. Values represent means ± 95% confidence intervals (CI).


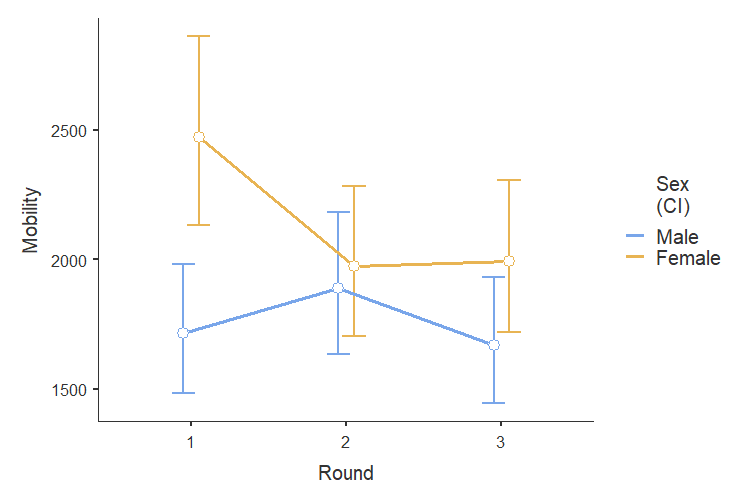


Supplementary Figure 6. Interaction effect between beetle sex and trial round on exploration. Values represent means ± 95% confidence intervals (CI).


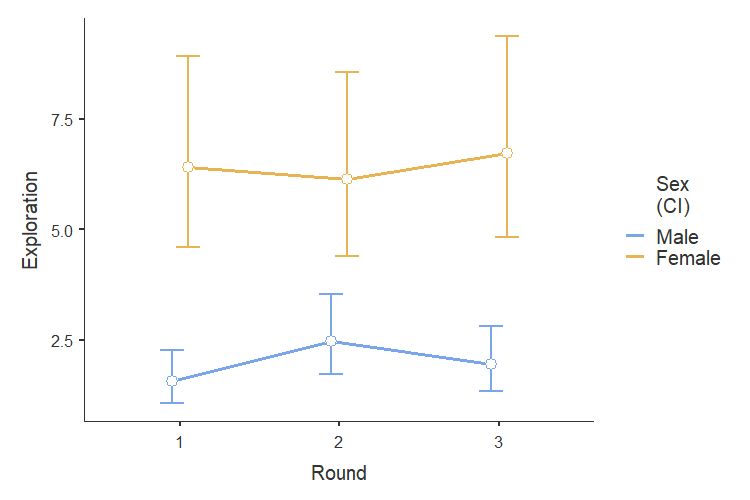

Supplement: Supplementary file 2 — Appendix S2 [file ECE3-15-e71718-s001.docx]
